# Adherence to treatment in children and adolescents with cystic fibrosis: a cross-sectional, multi-method study investigating the influence of beliefs about treatment and parental depressive symptoms

**DOI:** 10.1186/s12890-015-0038-7

**Published:** 2015-04-26

**Authors:** Nicola A Goodfellow, Ahmed F Hawwa, Alastair JM Reid, Rob Horne, Michael D Shields, James C McElnay

**Affiliations:** School of Pharmacy, Queen’s University Belfast, Medical Biology Centre, 97 Lisburn Road, Belfast, BT9 7BL, UK; Aston Pharmacy School, Aston University, Birmingham, B4 7ET, UK; Northern Ireland Paediatric Cystic Fibrosis Centre, Royal Belfast Hospital for Sick Children, Belfast, BT12 6BE, UK; UCL School of Pharmacy, BMA House, Tavistock Square, London, WC1H 9JP, UK; Centre for Infection and Immunity, Queen’s University Belfast, Belfast, UK; Department of Paediatrics, Royal Belfast Hospital for Sick Children, Belfast, BT12 6BE, UK

**Keywords:** Medication adherence, Cystic fibrosis, Parents, Adolescent, Child, Beliefs

## Abstract

**Background:**

Adherence to treatment is often reported to be low in children with cystic fibrosis. Adherence in cystic fibrosis is an important research area and more research is needed to better understand family barriers to adherence in order for clinicians to provide appropriate intervention. The aim of this study was to evaluate adherence to enzyme supplements, vitamins and chest physiotherapy in children with cystic fibrosis and to determine if any modifiable risk factors are associated with adherence.

**Methods:**

A sample of 100 children (≤18 years) with cystic fibrosis (44 male; median [range] 10.1 [0.2-18.6] years) and their parents were recruited to the study from the Northern Ireland Paediatric Cystic Fibrosis Centre. Adherence to enzyme supplements, vitamins and chest physiotherapy was assessed using a multi-method approach including; Medication Adherence Report Scale, pharmacy prescription refill data and general practitioner prescription issue data. Beliefs about treatments were assessed using refined versions of the Beliefs about Medicines Questionnaire-specific. Parental depressive symptoms were assessed using the Center for Epidemiologic Studies Depression Scale.

**Results:**

Using the multi-method approach 72% of children were classified as low-adherers to enzyme supplements, 59% low-adherers to vitamins and 49% low-adherers to chest physiotherapy. Variations in adherence were observed between measurement methods, treatments and respondents. Parental necessity beliefs and child age were significant independent predictors of child adherence to enzyme supplements and chest physiotherapy, but parental depressive symptoms were not found to be predictive of adherence.

**Conclusions:**

Child age and parental beliefs about treatments should be taken into account by clinicians when addressing adherence at routine clinic appointments. Low adherence is more likely to occur in older children, whereas, better adherence to cystic fibrosis therapies is more likely in children whose parents strongly believe the treatments are necessary. The necessity of treatments should be reinforced regularly to both parents and children.

**Electronic supplementary material:**

The online version of this article (doi:10.1186/s12890-015-0038-7) contains supplementary material, which is available to authorized users.

## Background

Adherence to treatment (or carrying out agreed healthcare recommendations) is approximately 50% in most chronic diseases and has been described as a “worldwide problem of striking magnitude” [1]. Cystic fibrosis is a complex, multi-system disease which presents both affected children and their parents with significant challenges regarding treatment management. Often the most effective treatments are complex and time consuming to administer [2]. Although children may be prescribed the most appropriate treatment and given appropriate advice, adherence to treatment recommendations in children with cystic fibrosis is reported to be below 50% [3]. Poor adherence has been linked to a decline in health outcomes such as pulmonary exacerbations, decline in baseline lung function and risk of hospitalisation [4,5]. Adherence to therapy is therefore essential to prevent worsening of the disease. Little is known about the extent of adherence to treatment in children with cystic fibrosis in the UK or indeed the factors affecting adherence in this population.

A factor that has been shown to account for a significant amount of the variance in adherence to treatment in several chronic diseases is beliefs about treatment [6,7]. It has been proposed that patients carry out an internal ‘cost-benefit’ analysis with regards to taking medicines, weighing up their perceived necessity and their concerns about the treatment [8]. High self-reported adherence to medications for chronic conditions in adults has been associated with high necessity beliefs and low concern beliefs using a necessity-concerns framework [6]. Equally, low necessity beliefs and high concerns about the potential harm of medication have been correlated with low adherence [6,9]. These treatment beliefs in relation to adherence have been underexplored in cystic fibrosis. One small study in adolescents with cystic fibrosis investigated beliefs about antibiotics, enzyme supplements and chest physiotherapy and found that doubts about necessity of antibiotics and chest physiotherapy were predictive of adherence [10]. Additionally, interviews with adolescents with cystic fibrosis and their parents identified that recognising the importance of therapies was a facilitator of adherence [11]. It is particularly pertinent to consider parental beliefs when considering chronic childhood diseases, where parents are responsible for administering or supervising their child’s therapy. Improved adherence to asthma treatment in children has been reported when their parents exhibit increased necessity-concern differentials (*i.e.* high necessity scores and low concern scores) [12].

Parental depression has previously been linked to poor adherence to treatment in children with asthma [13]. There is a paucity of data, on whether parental depressive symptoms influence adherence to treatment in children with cystic fibrosis in the UK. Recently there has been a focus in determining the prevalence of depressive symptoms present in children with cystic fibrosis and their caregivers through The International Depression/anxiety Epidemiology Study-CF (TIDES-CF) (www.tides-cf.org/) [14]. A significant association between depression present in mothers and an increase in child non-adherence to pancreatic enzyme supplements was reported in the US [15]. Similarly, Smith and Wood reported an association between parental depression and low adherence to chest physiotherapy in the US [16]. In contrast, a subsequent paper by Smith and colleagues, in 2010, reported that maternal depressive symptoms were positively related to adolescent adherence to chest physiotherapy [17]. This seemingly counter-intuitive finding was perceived to be due to either children being forced to become autonomous with respect to treatment or mothers putting excessive effort into airway clearance adherence and depressive symptoms manifesting in a subsequent ‘burn out’. Finally, a study conducted in Australia has shown that poor caregiver mental health was predictive of child non-adherence to chest physiotherapy [18].

Although studies have investigated cystic fibrosis treatment adherence in adults in the UK and both children and adults in the United States, there has been a lack of published research to date that has addressed adherence to treatment in children with cystic fibrosis in the UK. There are significant differences in healthcare provision and culture between the UK and USA; it therefore would be inaccurate to assume that factors relevant to adherence in the USA are the same as those affecting children with cystic fibrosis in the UK. Furthermore, no study has yet investigated parent-reported beliefs about treatments. The aim of the present study was to examine adherence to treatment in children with cystic fibrosis and determine if any modifiable risk factors, specifically beliefs about treatment and parental depressive symptoms, are associated with adherence. It was hypothesized that stronger necessity beliefs would result in increased treatment adherence whereas, stronger concern beliefs would result in lower treatment adherence. Additionally it was hypothesized that parental depressive symptoms would be associated with low adherence to treatment.

## Methods

### Participants

Favourable ethical opinion was granted by the Office for Research Ethics Northern Ireland (10/NIR02/42) and research governance approval was obtained from the Belfast Health and Social Care Trust (10095-AR-FC). A convenience sample of 100 children and their parents were consecutively recruited at the outpatient clinic of the Northern Ireland Paediatric Cystic Fibrosis Centre. Children (and their parents) were eligible for enrolment if the child had a consultant confirmed diagnosis of cystic fibrosis and was aged ≤18 years. Parents (and children) were not approached if the child had experienced a recent death in the family, if the child (≥6 years) was deemed by their doctor as not capable to provide assent or if the child was not accompanied to the clinic by a parent or guardian. Written consent was obtained from parents and assent obtained from children (≥6 years) after full explanation of the study. Throughout this paper the term child/children refers to children and young people 0-18 years old and the term parent describes the parents and/or guardians of these children.

Children were only included in the study once informed consent was obtained from their parent and assent was obtained as detailed above. Upon enrolment, baseline data were collected via interview and review of medical notes. Details recorded included sociodemographic data, healthcare provider details, medical history, current medication, additional therapies, clinical outcome data (weight, height, pulmonary function, Shwachman radiological score, bacterial colonisation status) and number of hospitalisations in the previous year. Following baseline data collection a series of questionnaires were administered to children and their parents by a research nurse or research pharmacist. The questionnaires were completed independently by parent and child at a time convenient to the running of the clinic.

### Measures

#### Composite adherence measurement

When measuring adherence it is recommended that a multi-method approach is employed, including both a self-report instrument and an objective measurement tool [1,3]. In the present study adherence levels to enzyme supplements, vitamins and chest physiotherapy were assessed using (a) scores from parent and/or child Medication Adherence Report Scale [19], (b) Patient Medication Records from each patient’s community pharmacist and (c) prescription records from the patient’s general practitioner (GP), as appropriate. A composite adherence measurement approach was used so that a child was designated as a low-adherer if they scored less than 80% in any one of the methods described. This dichotomous approach was employed to triangulate up to four different measurement methods of adherence. The threshold of 80% was chosen as this is the generally accepted lower limit of adherence adopted in a range of research articles after which point adherence may have a direct impact on patient outcomes [20].

- Medication Adherence Report Scale (MARS)
  Measuring self-reported non-adherence as opposed to self-reported adherence has been shown to correlate more closely to objective adherence measures such as pill counts [21]. The MARS questionnaire was used to measure the frequency of non-adherence to enzyme supplements, vitamins and chest physiotherapy with separate versions administered to parents and children (≥11 years). Each question was scored on a 1-5 Likert scale (always-never). Answers to each question were summed and transformed to range from 0-100, with higher scores indicating higher levels of self-reported adherence. If a parent stated that they were not responsible for treatment supervision/administration the parent MARS was not completed.
  Previously, when the MARS was administered to a sample of adolescents with cystic fibrosis to measure adherence to enzyme supplements, the internal consistency was low (α = 0.51) [10]. In an attempt to address this previously reported poor internal consistency with regards to enzyme supplements, one item was added and successfully piloted with 10 parents and children (≥11 years); ‘I/My child forgets to take them with snacks’, no further amendments were requested.
  The internal reliability, as measured by Cronbach’s alpha, for the MARS questionnaire for children was above 0.7 for each treatment. The internal reliability for the MARS parent scales were above 0.7 for vitamins and chest physiotherapy, however, the parent scale for enzyme supplements was low (α = 0.48). Principal Component Analysis (PCA) revealed that question 2, “*I alter the dose”*, from the parent MARS questionnaire for enzyme supplements loaded onto a separate component and had a low correlation with the other remaining questions on the scale. After removal of Question 2, the internal reliability improved (α = 0.60) and PCA indicated that the remaining items primarily loaded onto one component i.e. non-adherence. All analyses were therefore conducted after the removal of question 2 on the parent MARS for enzyme supplements.
- Community pharmacy Patient Medication Records (PMRs)
  Children were classified as low-adherers to enzyme supplements or vitamins if their community pharmacy records illustrated that they were dispensed less than 80% of the prescribed amounts during the previous 12 months. This was calculated using the Medication Refill Adherence (MRA) method recommended by Hess *et al*. [22] For patients who had an inpatient stay in hospital, during the 12 months prior to enrolment in the study, the length of stay was deducted from the total number of days evaluated to account for medication provided while in hospital [4]. Therefore, the revised calculation was:
  $$ \mathrm{M}\mathrm{R}\mathrm{A} = \frac{\mathrm{Total}\ \mathrm{days}\ \mathrm{supply}\ \mathrm{during}\ \mathrm{study}\ \mathrm{period}}{\mathrm{Total}\ \mathrm{number}\ \mathrm{o}\mathrm{f}\ \mathrm{days}\ \mathrm{evaluated}\ \hbox{--}\ \mathrm{Days}\ \mathrm{admitted}\ \mathrm{t}\mathrm{o}\ \mathrm{hospital}}\kern0.75em  \times 100\% $$
- General practitioner (GP) prescription data
  Similarly, patients were classified as low-adherers if the prescription issue records for enzyme supplements or vitamins disclosed by their GP indicated that less than 80% were issued in the previous 12 months using the MRA method described above.

### Beliefs about medicines questionnaire-specific (BMQ)

The BMQ-specific is a self-report questionnaire with separate versions for parents and children (≥11 years) to quantify beliefs in terms of necessity and concerns about treatment. Refined versions were administered separately for enzyme supplements, vitamins and chest physiotherapy. This scale has previously been administered to adolescents with cystic fibrosis where the need for further development of the concern scale for chest physiotherapy was recommended [10]. As such, three items were added to the concerns scale for chest physiotherapy *“I/My child finds chest physiotherapy tiring”*, *“I/My child finds it embarrassing to carry out chest physiotherapy”* and *“Chest physiotherapy makes me/my child feel worse”*. One additional item was included for all therapies, *“This treatment gives me/my child unpleasant side effects”*. These modifications were made during the study design phase in consultation with the author of the BMQ-specific and successfully piloted in 10 parents and their children (≥11 years) with no further amendments requested.

Due to the different numbers of questions depending on which therapy was being explored using the BMQ, the necessity and concern scores were summed for each participant and transformed to range from 0-100. High scores on each subscale indicated high necessity or concern beliefs respectively. The internal reliability, of the necessity and concern scales of the BMQ-specific for parents and children ≥11 years ranged from (0.75-0.90).

### Center for epidemiologic studies depression scale (CES-D)

The CES-D is a short 20-item validated scale to measure depressive symptoms in the general population [23]. In the present study the CES-D was administered to parents. A cut-off score of ≥16 was used to establish whether parents were experiencing clinically relevant depressive symptoms [23]. The CES-D, in preference to the Hospital Anxiety and Depression Scale, was chosen to prevent underestimation of depressive symptoms present in parents of children with cystic fibrosis [24].

The order of questionnaire administration remained consistent throughout the study (BMQ, MARS, CES-D). Researchers administering questionnaires were independent of the clinicians and this was stressed to reduce any perceived pressure of social desirability.

### Data analysis

All analyses were conducted using IBM SPSS Statistics (version 19, SPSS Inc, USA). Group differences (high-adherers and low-adherers) were examined using t-tests or the Mann Whitney U test, as appropriate for continuous variables, *e.g.* beliefs about medicines. Group differences for categorical variables were examined using Chi-square analysis or the Fisher’s Exact test, as appropriate. The significance level was set at 0.05 throughout.

Due to the large number of variables investigated and comparatively low number of high-adherence observations, only those factors which were shown to have significant influences on adherence or had a p value of p < 0.2 were subjected to multivariate analysis using logistic regression. Separate analyses were conducted for adherence to enzyme supplements (n = 80), vitamins (n = 88) and chest physiotherapy (n = 98).

The validity of the refined MARS and BMQ-specific were assessed using Principal Component Analysis on IBM SPSS Statistics (version 19, SPSS Inc, USA). Internal reliability of all questionnaires was quantified using Cronbach’s alpha, for each dimension.

## Results

**Figure 1:**
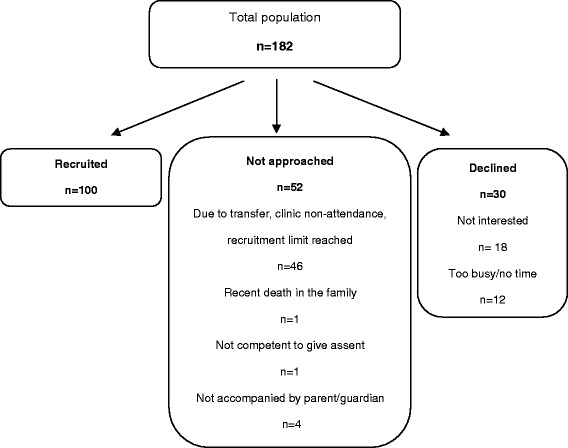
Participant enrolment.

A total of 100 participants were enrolled during the study (Figure 1). The baseline characteristics of participants are shown in Table 1. Children who did not participate in the study did not differ significantly in terms of age, gender, BMI percentile or *P.aeruginosa* colonisation status. Non-participants did, however, have significantly higher FEV_1_% predicted when compared to participants (p < 0.01). The majority of parents recruited to the study were mothers (83%). Enzyme supplements were prescribed for 81 children, vitamins were prescribed for 98 children and chest physiotherapy was prescribed for 99 children.

**Table 1:** **Baseline characteristics of participants**

**Child characteristics**	**Participants**
**Age of child (years)**	n = 100
Median (range)	10.1 (0.2-18.6)
**Gender**	n = 100
% male	44
**Ethnicity**	n = 100
% Caucasian	100
**FEV**_**1**_ **% predicted** (in children over 6 years)	n = 71
FEV_1_ ≥ 70% (mild disease)	57 (80.3%)
FEV_1_ 40-69% (moderate disease)	11 (15.5%)
FEV_1_ ≤ 39% (severe disease)	3 (4.2%)
Mean FEV_1_% predicted (±95% C.I.)	84% (±4.54)
**BMI percentile** (children <18 years)	n = 98
<15^th^ percentile	10 (10.2%)
≥15^th^ percentile	88 (89.8%)
**BMI** (≥18 years)	n = 2
≥18.5 ≤ 25	2 (100%)
**Swachman-Kulczycki radiological score/25**	
Median (IQR)	22 (20-23)
**Colonisation status**	n = 100
*B.cepacia*	5 (5% chronic)
Methicillin-resistant *Staphylococcus aureus* (MRSA)	7 (7% chronic)
*P.aeruginosa* ^†^	3 (3% intermittent)
	12 (12% chronic)
**Days in hospital over the previous year**	n = 100
Median (IQR)	0 (0-1.75)
**Number of medications currently prescribed**	n = 100
Median (IQR)	6 (4-8)
**Enzyme supplement prescribed** [n = %]	81 (81%)
**Vitamins prescribed** [n = %]	98 (98%)
**Chest physiotherapy prescribed** [n = %]	99 (99%)
**Other medical diagnosis**	
No	84 (84%)
Yes	16 (16%)

^†^ *P.aeruginosa* classified as chronic using the Leeds criteria. *FEV*_1_ forced expiratory volume in 1 s; *BMI* body mass index; *IQR* inter-quartile range.

**Figure 2:**
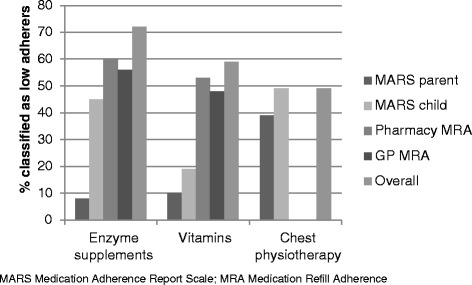
Classification of low-adherence using a multi-method approach.

Adherence to treatment varied depending on the adherence measurement method used, treatment type and respondent (i.e. parent or child) (Figure 2). Using the composite adherence measurement approach 58 (72%) were classified as low-adherers to enzyme supplements, 58 children (59%) were classified as low-adherers to vitamins and 48 (49%) were classified as low-adherers to chest physiotherapy (Figure 2). Seventy nine participants were prescribed all three treatments, the number of participants classified as high adherers to all treatments was 14, whereas, 25 were classified as low adherers to all treatments. Children ≥11 years were significantly more likely to report low-adherence to enzyme supplements than their parents (p < 0.01).

**Figure 3:**
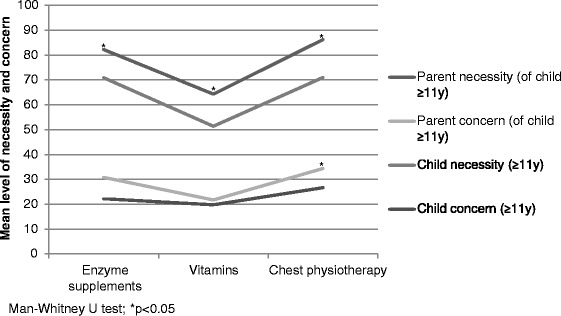
Graph illustrating beliefs about treatments in a subset of children (≥11 years) and their parents.

Beliefs about treatments varied across treatments and respondents. Concern beliefs about treatment were low for all three treatments, particularly vitamins. Parents and children reported high necessity beliefs for enzyme supplements and chest physiotherapy. Necessity beliefs for vitamins were lower by comparison. In a subgroup of children 11-18 years and their parents (who both completed the BMQ) it was revealed that there were significant differences in the strength of necessity beliefs with parents having significantly stronger necessity beliefs for enzyme supplements (n = 66; p = 0.03), vitamins (n = 86; p < 0.01) and chest physiotherapy (n = 86; p < 0.01) than their children. Similarly, parents were shown to have higher concerns about chest physiotherapy than their children (n = 86; p = 0.04) (Figure 3).

Clinically relevant depressive symptoms present in parents, as measured by the CES-D, occurred in 21 parents (23.3%). Approximately half of these parents (n = 10) reported scores ≥27 which can be indicative of major depression [25]. There were missing data for 5 parents and 5 parents declined to complete this questionnaire.

Full details of the univariate analysis for each treatment are presented in an Additional file 1. A summary of factors showing significance or those which had a p value of p < 0.2 are presented in Table 2.

**Table 2:** **Summary of group differences (for variables with p < 0.2) between high adherers and low adherers**

**Treatment**	**Parameter**	**High- adherers**	**Low-adherers**	**p**
**Enzyme supplements**	**Age**	n = 23	n = 58	
	Median (IQR)	6 (2-10)	11.5 (7.5-15)	≤0.001*
	**Colonisation status**			
	n (%)			
	*P.*aeruginosa			
	Chronic (n = 11)	0 (0)	11 (19)	
	Not chronic (n = 70)	23 (100)	47 (81)	0.029*
	**BMQ (parent)**	n = 23	n = 58	
	median (IQR)			
	Necessity	85 (80-100)	80 (65-96)	0.129^§^
	Concern	21 (13-38)	29 (17-42)	0.073^§^
	**BMQ (child)**	n = 2	n = 31	
	median (IQR)			
	Concern	6 (N/A)	21 (13-33)	0.120^§^
**Vitamins**	**Age**	n = 40	n = 58	0.074^§^
	Median (IQR)	9 (3-12)	12 (6-15)	
	**Colonisation status**			
	n (%)			
	*P.aeruginosa*			
	Chronic (n = 12)	2 (5)	10 (17)	
	Not chronic (n = 1)	38 (95)	48 (83)	0.115^§^
	**Presence of concurrent medical condition**			
	n (%)			
	Yes (n = 15)	9 (23)	6 (10)	
	No (n = 83)	31 (78)	52 (90)	0.175^§^
	**BMQ (parent)**	n = 40	n = 58	
	median (IQR)			
	Concern	17 (4-25)	21 (8-33)	0.117^§^
	**CES-D (parent)**	n = 37	n = 51	
	Median (IQR)	6 (3-14)	11 (5-15)	0.073^§^
**Chest physiotherapy**	**Age**	n = 50	n = 48	≤0.001*
	Median (IQR)	7 (2-11)	13 (8-15)	
	**Number of medications currently prescribed**	n = 50	n = 48	
	Median (IQR)	6 (5-8)	5 (4-8)	0.080^§^
	**Presence of concurrent medical condition**			
	n (%)			
	Yes (n = 16)	4 (8)	12 (25)	
	No (n = 82)	46 (92)	36 (75)	0.045*
	**BMQ (parent)**	n = 50	n = 48	
	median (IQR)			
	Necessity	95 (85-100)	80 (70-95)	0.001*
	Concern	25 (22-33)	33 (23-47)	0.032*

^§^p < 0.2; *p < 0.05; *IQR* inter-quartile range; *BMQ* Beliefs about Medicines Questionnaire; CES-D Centre for Epidemiologic Studies Depression Scale.

Full results available in Additional file 1.

A preliminary effects model was constructed using those variables that had a p value of p < 0.20 in the univariate analysis. The preliminary effects model for enzyme supplements included; increasing age, increasing parental BMQ necessity score and increasing parental BMQ concern score. The preliminary effects model for vitamins included; increasing age, presence of concurrent medical condition, increasing parental BMQ concern score, increasing CES-D score. The preliminary effects model for chest physiotherapy included; increasing age, number of medications, presence of concurrent medical condition, increasing parental BMQ necessity score and increasing parental BMQ concern score. Bacterial colonisation status and child scores on the BMQ were not included in the logistic regression analyses as the low number in the high-adherence group precluded analysis. A backward stepwise logistic regression analysis was then performed on the preliminary effects models. The resulting main effects models for enzyme supplements and chest physiotherapy are presented in Tables 3 and 4. No main effects model is presented for vitamins as none of the variables accounted for an independent statistically significant contribution to predicting adherence to vitamins. Based on these models, child age and parental necessity beliefs were predictive of adherence to enzyme supplements and chest physiotherapy. Advancing age in children, was associated with a decreased likelihood of adherence to enzyme supplements (OR = 0.79, 0.70-0.90) and chest physiotherapy (OR = 0.82, 0.74-0.90) whereas, increased parental necessity beliefs was associated with improved adherence to these therapies (OR = 1.05 each). Parental depressive symptoms were not found to be predictive of adherence to enzyme supplements, vitamins or chest physiotherapy in the population studied.

**Table 3:** **Main effects model for high adherence to enzyme supplements**

**Independent variable**	**B**	**SE**	**Odds ratio (OR)**	**95% CI of OR**
Increasing age (in years)	-0.23	0.06	0.79*	0.70-0.90
Increasing parental BMQ necessity score	0.05	0.02	1.05*	1.01-1.09

B regression coefficient; *SE* standard error associated with the coefficient B; *p < 0.05; n = 80 (1 case removed due to outlier); *BMQ* Beliefs about Medicines Questionnaire. The model explained between 23.9% (Cox and Snell R Square) and 34.5% (Nagelkerke R square) of the variance in adherence status.

**Table 4:** **Main effects model for high adherence to chest physiotherapy**

**Independent variable**	**B**	**SE**	**Odds ratio**	**95% CI**
Increasing age (in years)	-0.20	0.05	0.82*	0.74-0.90
Increasing parental BMQ necessity score	0.05	0.02	1.05*	1.02-1.09

B regression coefficient; SE standard error associated with the coefficient B; *p < 0.05; *p < 0.01; n = 98 (1 case had missing data); BMQ Beliefs about Medicines Questionnaire. The model explained between 27.1% (Cox and Snell R square) and 36.2% (Nagelkerke R square) of the variance in adherence status.

## Discussion

In the current study 55% children (≥11 years) and 92% parents indicated high-adherence to enzyme supplements using self-report methods. This contrasts with previous studies in children using self-report which indicated that adherence to enzyme supplements lies between 77-98% [3,26,27]. Overall only 28% of participants were classified as high-adherers when all methods (including prescription refill and prescription issue data) were taken into account. This rate of adherence is comparable to diary data (27%) observed in studies in the USA [3]. With regards to vitamins, self-reported adherence was high (90% parents; 81% children ≥11 years) and similar to the range illustrated by previous studies using self-report measures [3]. Using the multi-method approach described, 41% of children were classified as high-adherers to vitamins which is comparable to the proportion of children classified as adherent in a US study investigating pharmacy refill adherence and diary data (34% and 22% respectively) [3]. To assess adherence to chest physiotherapy only parent and child self-reports were assessed as there were no objective measures available. Nevertheless, 51% of children (≥11 years) and 61% parents reported adherence to chest physiotherapy which fell within the expected range for this method of measurement [3,27].

In order to reduce bias from one particular adherence measurement method, a multi-method approach was employed to assess adherence in the current study. Previously, overestimation of adherence by 25.3% to nebulised therapy using self-report was reported when compared to electronic monitoring [28]. Overestimation of adherence using self-report was also evidenced in the present study in comparison to prescription data obtained for enzyme supplements and vitamins. When children (≥11 years) used the self-report measure, their responses were closer to non-adherence measured via the objective pharmacy and GP prescription data when compared with their parents. This difference has not previously been reported in the literature and this could occur because parents are more aware of socially desirable responses or that parents believe that their children are taking their medication when in fact they are not.

This is the first study to investigate the influence of parental beliefs and depressive symptoms on child adherence to therapy in cystic fibrosis. Parental beliefs about the necessity of enzyme supplements and chest physiotherapy were found to be predictive of their child’s adherence to these therapies. For every percentage increase in score children were 5% more likely to be adherent to enzyme supplements and chest physiotherapy. Children whose parents had reported high necessity beliefs regarding their child’s use of enzyme supplements or chest physiotherapy were significantly more likely to be classified as high-adherers to these treatments. It has been reported previously that poor adherence to nebulised therapy in adults was associated with doubts about nebuliser necessity and similarly, non-adherence to antibiotics and chest physiotherapy has been related to adolescents’ doubts about their necessity in cystic fibrosis [10,26]. In the present study, necessity beliefs for all treatments were significantly higher for parents of children 11-18 years than those reported by their children. One reason for this may be that children have inconsistent treatment goals compared to those of parents and physicians [29]. The finding that children may perceive necessity of treatment differently to their parents could be of particular importance when children become autonomous and have more responsibility for their treatments.

The finding that vitamins received lower necessity and concern scores compared with enzyme supplements and chest physiotherapy indicates a lack of understanding surrounding the importance of vitamin supplementation in children with cystic fibrosis. Vitamins are perhaps not considered ‘real’ medicines by parents and children since they are widely available without prescription. This could explain why parental beliefs about vitamins were not predictive of their child’s adherence to this treatment. Concern beliefs were not significant independent predictors of treatment adherence, however, this could have been due to the low levels of concern expressed about the treatments in this sample.

In the present study approximately a quarter (23.3%) of parents reported symptoms indicative of depression. This corresponds to data reported from the UK arm of the TIDES-CF study which indicated that 28.3% of parents reported clinically relevant levels of depressive symptoms [30]. Additionally, caregiver rates of depression have been found to vary significantly between maternal and paternal responses to the CES-D with mothers reporting significantly more depressive symptoms, i.e. 35% compared with 25% [14]. In this study parental depressive symptoms were not found to be independently predictive of child adherence to enzyme supplements, vitamins or chest physiotherapy, therefore, no evidence was found to support the use of routine monitoring of parental depressive symptoms as an indicator for poor treatment adherence in their children within the present study population. This contrasts with previous reports indicating parental depressive symptoms were associated with enzyme supplement and chest physiotherapy adherence in children [15-18].

Child age was also an important adherence indicator; for each advancing year, adherence to enzyme supplements and chest physiotherapy was reduced by approximately 20%. This observation that advancing age reduces adherence to therapy in cystic fibrosis supports previous European findings [27]. Barriers to treatment adherence specifically reported by adolescents include time pressures, competing priorities, privacy concerns and lack of consequences of treatment non-adherence [11].

### Limitations

When monitoring adherence to treatment, each measurement method has integral limitations, however, this was minimised by adopting a multi-method approach to measurement. Additionally, due to the nature of cross-sectional study design, causality could not be determined. Insufficient sample size precluded analysis of bacterial colonisation and BMQ child responses between adherent and non-adherent groups. Pharmacy records are not a closed system, however, all pharmacies identified by parents were contacted and patient medication records received. There was the potential for selection bias to occur with those who did not participate potentially being less adherent to treatment, therefore the adherence levels reported in this article should be regarded as conservative estimates.

## Conclusions

Low adherence to enzyme supplements, vitamins and chest physiotherapy was revealed. Future research should focus on why there is a significant difference between parent and child beliefs about treatments and on the process of parental transfer of beliefs about treatment. Future adherence interventions addressing parental beliefs about enzyme supplements and chest physiotherapy may improve child adherence to treatment. There is a larger scope to improve adherence if interventions are targeted at older children.

## Additional file

Additional file 1:
**Full univariate analyses for adherence to enzyme supplements, vitamins and chest physiotherapy.**
