# Supplementary material for: Adherence to treatment in children and adolescents with cystic fibrosis: a cross-sectional, multi-method study investigating the influence of beliefs about treatment and parental depressive symptoms
Source: BMC Pulm Med. 2015 Apr 26;15:43. doi: 10.1186/s12890-015-0038-7 (PMC4417214; doi:10.1186/s12890-015-0038-7)
Supplement: Additional file 1: — Full univariate analyses for adherence to enzyme supplements, vitamins and chest physiotherapy. [file 12890_2015_38_MOESM1_ESM.docx]

| **Univariate analyses for adherence to enzyme supplements** | | | |
| --- | --- | --- | --- |
|  | **High adherers** | **Low adherers** | **p** |
| **Age**  Median (IQR) | n=23  6 (2-10) | n=58  11.5 (7.5-15) | 0.000***** |
| **Gender**  n (%)  Male (n=38)  Female (n=43) | 10 (44)  13 (57) | 28 (48)  30 (52) | 0.886 |
| **FEV_1_% predicted**  Mean (SD) | n=12  81 (21) | n=46  83 (20) | 0.780 |
| **Healthy growth indicator**  (BMI centile ≥15^th^ or BMI ≥18.5≤25)  n (%)  Healthy (n=72)  Unhealthy (n=9) | 21 (91)  2 (9) | 51 (88)  7 (12) | 1.000 |
| **Swachman-Kulczycki radiological score (/25)**  Median (IQR) | n=23  22 (20-23) | n=58  22 (19-23) | 0.739 |
| **Colonisation status**  n (%)   - *B.cepacia*   Chronic (n=4)  Clear (n=77)   - *MRSA*   Chronic (n=7)  Intermittent (n=2)  Clear (n=72)   - *P.aeruginosa*   Chronic (n=11)  Non-chronic (n=70) | 0 (0)  23 (100)  2 (9)  0 (0)  21 (91)  0 (0)  23 (100) | 4 (7)  54 (93)  5 (9)  2 (3)  51 (88)  11 (19)  47 (81) | 0.573  1.000  0.029* |
| **Days in hospital over the previous year**  Median (IQR) | n=23  0 (0-4) | n=58  0 (0-1.5) | 0.815 |
| **Number of medications currently prescribed**  Median (IQR) | n=23  6 (6-8) | n=58  7 (5-8) | 0.622 |
| **Presence of concurrent medical condition**  n (%)  Yes (n=12)  No (n=69) | 4 (17)  19 (83) | 8 (14)  50 (86) | 0.733 |
| **Parent education attainment**  n (%)  Secondary (n=50)  Tertiary (n=28)  Other (n=3) | 13 (57)  9 (39)  1 (4) | 37 (64)  19 (33)  2 (3) | 0.825 |
| **BMQ (parent)**  median (IQR)  Necessity  Concern | n=23  85 (80-100)  21 (13-38) | n=58  80 (65-96)  29 (17-42) | 0.129^§^  0.073^§^ |
| **BMQ (child)**  median (IQR)  Necessity  Concern | n=2  83 (NA)  6 (N/A) | n=31  70 (60-85)  21 (13-33) | 0.425  0.120^§^ |
| **CES-D (parent)**  Median (IQR) | n=21  8 (4-13) | n=53  8 (5-15) | 0.593 |
| **CES-D (parent) dichotomised score**  n (%)  ≥16 (n=16)  <16 (n=58) | 3 (14)  18 (86) | 13 (25)  40 (76) | 0.532 |

^§p<0.2; *p<0.05; n% may add up to >100% due to rounding; N/A not applicable; FEV1 Forced expiratory Volume in 1s; IQR inter-quartile range; BMQ Beliefs about Medicines Questionnaire; CES-D Centre for Epidemiologic Studies Depression Scale.^

| **Univariate analyses for adherence to vitamins** | | | |
| --- | --- | --- | --- |
|  | **High adherers** | **Low adherers** | **p** |
| **Age**  Median (IQR) | n=40  9 (3-12) | n=58  12 (6-15) | 0.074^§^ |
| **Gender**  n (%)  Male (n=43)  Female (n=55) | 15 (38)  25 (63) | 28 (48)  30 (52) | 0.396 |
| **FEV_1_% predicted**  Mean (SD) | n=26  84 (20) | n=45  84 (19) | 0.964 |
| **Healthy growth indicator**  (BMI centile ≥15^th^ or BMI ≥18.5≤25)  n (%)  Healthy (n=87)  Unhealthy (n=11) | 37 (93)  3 (8) | 50 (86)  8 (14) | 0.517 |
| **Swachman-Kulczycki radiological score (/25)**  Median (IQR) | n=40  22 (21-23) | n=58  22 (19-23) | 0.679 |
| **Colonisation status**  n (%)   - *B.cepacia*   Chronic  Clear   - *MRSA*   Chronic (n=7)  Intermittent (n=3)  Clear (n=88)   - *P.aeruginosa*   Chronic (n=12)  Not chronic (n=86) | 3 (8)  37 (93)  4 (10)  1 (3)  35 (88)  2 (5)  38 (95) | 2 (3)  56 (97)  3 (5)  2 (3)  53 (91)  10 (17)  48 (83) | 0.396  0.757  0.115^§^ |
| **Days in hospital over the previous year**  Median (IQR) | n=40  0 (0-2) | n=58  0 (0-3) | 0.602 |
| **Number of medications currently prescribed**  Median (IQR) | n=40  6 (5-8) | n=58  6 (4-8) | 0.454 |
| **Presence of concurrent medical conditions**  n (%)  Yes (n=15)  No (n=83) | 9 (23)  31 (78) | 6 (10)  52 (90) | 0.175^§^ |
| **Parent education attainment**  n (%)  Secondary (n=60)  Tertiary (n=33)  Other (n=5) | 21 (53)  16 (40)  3 (8) | 39 (67)  17 (29)  2 (3) | 0.314 |
| **BMQ (parent)**  median (IQR)  Necessity  Concern | n=40  65 (51-75)  17 (4-25) | n=58  65 (54-75)  21 (8-33) | 0.409  0.117^§^ |
| **BMQ (child)**  median (IQR)  Necessity  Concern | n=13  55 (40-75)  17 (2-23) | n=30  48 (40-58)  17 (8-38) | 0.331  0.276 |
| **CES-D (parent)**  Median (IQR) | n=37  6 (3-14) | n=51  11 (5-15) | 0.073^§^ |
| **CES-D (parent) dichotomised score**  n (%)  ≥16 (n=20)  <16 (n=68) | 8 (22)  29 (78) | 12 (24)  39 (77) | 1.000 |

^§p<0.2; *p<0.05; n% may add up to >100% due to rounding; FEV1 Forced expiratory Volume in 1s; IQR inter-quartile range; BMQ Beliefs about Medicines Questionnaire; CES-D Centre for Epidemiologic Studies Depression Scale.^

| **Univariate analyses for adherence to chest physiotherapy** | | | |
| --- | --- | --- | --- |
|  | **High adherers** | **Low adherers** | **p** |
| **Age**  Median (IQR) | n=50  7 (2-11) | n=48  13 (8-15) | 0.000* |
| **Gender**  n (%)  Male (n=44)  Female (n=54) | 24 (48)  26 (52) | 20 (42)  28 (58) | 0.669 |
| **FEV_1_% predicted**  Mean (SD) | n=28  81 (17) | n=42  86 (21) | 0.314 |
| **Healthy growth indicator**  (BMI centile ≥15^th^ or BMI ≥18.5≤25)  n (%)  Healthy (n=87)  Unhealthy (n=11) | 46 (92)  4 (8) | 41 (85)  7 (15) | 0.476 |
| **Swachman-Kulczycki radiological score (/25)**  Median (IQR) | n=50  22 (20-23) | n=48  22 (19-23) | 0.592 |
| **Colonisation status**  n (%)   - *B.cepacia*   Chronic (n=5)  Clear (n=93)   - *MRSA*   Chronic (n=7)  Intermittent (n=3)  Clear (n=88)   - *P.aeruginosa*   Chronic (n=12)  Not chronic (n=86) | 2 (4)  48 (96)  5 (10)  2 (4)  43 (86)  4 (8)  46 (92) | 3 (6)  45 (94)  2 (4)  1 (2)  45 (94)  8 (17)  40 (83) | 0.674  0.507  0.317 |
| **Days in hospital over the previous year**  Median (IQR) | n=50  0 (0-0) | n=48  0 (0-9) | 0.217 |
| **Number of medications currently prescribed**  Median (IQR) | n=50  6 (5-8) | n=48  5 (4-8) | 0.080^§^ |
| **Presence of concurrent medical condition**  n (%)  Yes (n=16)  No (n=82) | 4 (8)  46 (92) | 12 (25)  36 (75) | 0.045* |
| **Parent education attainment**  n (%)  Secondary (n=61)  Tertiary (n=32)  Other (n=5) | 30 (60)  17 (34)  3 (6) | 31 (65)  15 (31)  2 (4) | 0.944 |
| **BMQ (parent)**  median (IQR)  Necessity  Concern | n=50  95 (85-100)  25 (22-33) | n=48  80 (70-95)  33 (23-47) | 0.001*  0.032* |
| **BMQ (child)**  median (IQR)  Necessity  Concern | n=12  80 (51-80)  26 (11-40) | n=31  80 (55-90)  22 (17-39) | 0.462  0.967 |
| **CES-D (parent)**  Median (IQR) | n=46  8 (4-14) | n=43  9 (5-18) | 0.383 |
| **CES-D (parent) dichotomised score**  n (%)  ≥16 (n=21)  <16 (n=68) | 8 (17)  38 (83) | 13 (30)  30 (70) | 0.240 |

^§p<0.2; *p<0.05; n% may add up to >100% due to rounding; FEV1 Forced expiratory Volume in 1s; IQR inter-quartile range; BMQ Beliefs about Medicines Questionnaire; CES-D Centre for Epidemiologic Studies Depression Scale.^
